# Effect of Preventive Supplementation with Zinc and Other Micronutrients on Non-Malarial Morbidity in Tanzanian Pre-School Children: A Randomized Trial

**DOI:** 10.1371/journal.pone.0041630

**Published:** 2012-08-03

**Authors:** Jacobien Veenemans, Laura R. A. Schouten, Maarten J. Ottenhof, Theo G. Mank, Donald R. A. Uges, Erasto V. Mbugi, Ayşe Y. Demir, Rob J. Kraaijenhagen, Huub F. J. Savelkoul, Hans Verhoef

**Affiliations:** 1 Cell Biology and Immunology Group, Wageningen University, Wageningen, The Netherlands; 2 Laboratory for Microbiology and Infection Control, Amphia Hospital, Breda, The Netherlands; 3 Department of Parasitology, Public Health Laboratory, Haarlem, The Netherlands; 4 Laboratory for Clinical and Forensic Toxicology and Drug Analysis, Department of Pharmacy, University Medical Center, University of Groningen, Groningen, The Netherlands; 5 Muhimbili University of Health and Allied Sciences, Dar es Salaam, Tanzania; 6 Laboratory for Clinical Chemistry and Haematology, Meander Medical Centre, Amersfoort, The Netherlands; 7 MRC International Nutrition Group, London School of Hygiene and Tropical Medicine, London, United Kingdom; Tulane University, United States of America

## Abstract

**Background:**

The efficacy of preventive zinc supplementation against diarrhea and respiratory illness may depend on simultaneous supplementation with other micronutrients. We aimed to assess the effect of supplementation with zinc and multiple micronutrients on diarrhea and other causes of non-malarial morbidity.

**Methods and Findings:**

Rural Tanzanian children (n = 612) aged 6–60 months and with height-for-age z-score < –1.5 SD were randomized to daily supplementation with zinc (10 mg) alone, multi-nutrients without zinc, multi-nutrients with zinc, or placebo. Children were followed for an average of 45 weeks. During follow-up, we recorded morbidity episodes. We found no evidence that concurrent supplementation with multi-nutrients influenced the magnitude of the effect of zinc on rates of diarrhea, respiratory illness, fever without localizing signs, or other illness (guardian-reported illness with symptoms involving skin, ears, eyes and abscesses, but excluding trauma or burns). Zinc supplementation reduced the hazard rate of diarrhea by 24% (4%–40%). By contrast, multi-nutrients seemed to increase this rate (HR; 95% CI: 1.19; 0.94–1.50), particularly in children with asymptomatic *Giardia* infection at baseline (2.03; 1.24–3.32). Zinc also protected against episodes of fever without localizing signs (0.75; 0.57–0.96), but we found no evidence that it reduced the overall number of clinic visits.

**Conclusions:**

We found no evidence that the efficacy of zinc supplements in reducing diarrhea rates is enhanced by concurrent supplementation with other micronutrients. By reducing rates of fever without localizing signs, supplementation with zinc may reduce inappropriate drug use with anti-malarial medications and antibiotics.

**Trial Registration:**

ClinicalTrials.gov NCT00623857

## Introduction

There are no policy recommendations for preventive zinc interventions in developing countries, despite evidence from a recent meta-analysis of trials, mostly conducted in Asia and Latin America, that it can reduce the overall rates of diarrhea and respiratory illnesses by 20% and 14%, and rates of mortality by 6%. Results have varied between trials, however, probably due to heterogeneity in study populations with respect to factors that determine the response to zinc [1].

Zinc is required for DNA synthesis, RNA transcription, cell division, cell activation and other basic biological functions at the cellular level [2], and possibly for the functioning of tight junctions. Zinc plays a critical role in maintaining epithelial barrier function [3]. Its deficiency has been reported to reduce epithelial barrier function [3], and to impair the development and functioning of cells that mediate both innate and acquired immunity. In addition, zinc possibly also has anti-inflammatory properties [2].

Zinc deficiency often coexists with other nutritional deficiencies, and simultaneous supplementation of multiple micronutrients may be required to overcome the lack of response that can be found when zinc is given alone [4]. In a recent review of steps to be taken to translate research into zinc intervention programs, the International Zinc Nutrition Consultative Group urged the implementation of studies to assess the efficacy of zinc in combination with other micronutrients [5].

Few trials have been conducted in Africa, where the etiology of disease and environmental factors such as diet and micronutrient deficiencies may be different from Asia and Latin America.

In this study, we aimed to assess the effect of supplementation with zinc and multiple micronutrients on diarrhea and other causes of non-malarial morbidity in young, rural Tanzanian children. In addition, we explored baseline factors that determined the magnitude of the morbidity response to such supplementation. These baseline factors included infection with *Giardia intestinalis*, because its role as diarrhea-causing agent is controversial: some studies reported an association with acute and persistent diarrhea, whereas others found no association, or even that *Giardia* infection was associated with protection against acute diarrhea [6].

## Methods

The protocol for this trial and supporting CONSORT checklist are available as supporting information; see Checklist S1 and Protocol S1. There were no important changes to methods after trial commencement.

### Ethics Statement

The study was approved by ethical review committees in The Netherlands and Tanzania (National Health Research Ethics Review sub-Committee). It was conducted in accordance with guidelines expressed in the Declaration of Helsinki. We sought and obtained written individual informed consent for all children; parents or guardians were invited to sign (or thumbprint if illiterate) the informed consent form in the presence of a member of the community as impartial witness (who countersigned the form).

### Study Area

The study was part of a trial (ClinicalTrials.gov: NCT00623857) with the primary aim to assess the effects of supplementation with zinc and other micronutrients on malaria rates. Details of study design and effects on malaria rates are reported elsewhere [3]. The trial was performed in four villages (Ngojoro, Kwangwe, Bondo and Kwadoya) near Segera (S 05°23.050, E 038°34.745) in Handeni District, north-eastern Tanzania. This rural area is highly endemic for malaria, and access to health care is limited, with no primary care facilities in the study area except the research dispensary that was constructed specifically for this study. The vast majority of families live in self-constructed clay houses, few with adjacent pit-latrines. Most households are self-sustaining and the dietary intake of the children restricted to maize and beans, with very low intake from animal products. This food contains a high concentration of phytates that limits the absorption of several trace elements, including zinc.

### Recruitment

Data were collected between February 2008 and March 2009. All resident children aged 6–60 months were invited for screening. Venous blood and fresh stool samples were collected, anthropometric measurements recorded in duplicate, and children were examined by a clinical officer. Children were eligible when having a height-for-age z-score ≤ –1.5 SD (which tends to select for zinc deficiency) [7], weight-for-height z-score > –3 SD (thus excluding severely wasted children for ethical reasons), no signs of severe chronic diseases, and hemoglobin concentration >70 g/L. Children whose parents refused consent or intended to move outside the study area during the follow-up period were excluded.

### Randomization and Interventions

Children were randomized within 6 strata defined by malaria infection (binary) and age class (6–17 months, 18–35 months and 36–60 months) and randomly permuted blocks with size randomly selected of 4 or 8. A colleague not otherwise involved in the trial used tables with random numbers to generate the allocation sequence. Children received daily supplements with either zinc alone (10 mg as gluconate), multi-nutrients without zinc, zinc combined with multi-nutrients or placebo. The levels of magnesium and vitamin C in the multi-nutrient supplement were below upper limits that were based on osmotic diarrhea and related gastrointestinal disturbances as critical endpoints [8]; further details about the composition of the multi-nutrient supplement are shown in Table 1. Supplements were packed as transparent blister-strips, each containing 15 capsules with powder that was similar in taste and appearance for all 4 intervention groups. Capsules were color-coded to reduce the chance that children would receive the wrong supplement. The color code was not disclosed to the researchers until after the database had been finalized.

**Table 1:** Target dose and form of multi-nutrient supplement (including zinc).

Active substance	Target level			Form	Infants 6–12 months		Children 1–3 years		Children 4–5 years	
	Declared	Overage *	Formulated		RNI	UL	RNI	UL	RNI	UL
Vitamin A	300 µg RAE	50%	450 µg RAE†	All-*trans* retinyl acetate (powder)	400 µg	600 µg	400 µg	600 µg	450 µg	900 µg
Vitamin B_1_	0.5 mg	25%	0.625 mg	Thiamin mononitrate	0.3 mg	ND	0.5 mg	ND	0.6 mg	ND
Vitamin B_2_	0.5 mg	10%	0.55 mg	Riboflavin	0.4 mg	ND	0.5 mg	ND	0.6 mg	ND
Niacin	6 mg NE	10%	6.6 mg	Niacin	4 NE	ND	6 NE	10 mg	8 NE	15 mg
Vitamin B_6_	0.5 mg	15%	0.575 mg	Pyridoxine	0.3 mg	ND	0.5 mg	30 mg	0.6 mg	40 mg
Folate	150 µg DFE	25%	93.75 µg‡	Folic acid	80 µg	ND	160 µg	300 µg DFE	200 µg	400 µg DFE
Vitamin B_12_	0.9 µg	30%	1.17 µg	Cyanocobalamid in mannitol	0.5 µg	ND	0.9 µg	ND	1.2 µg	ND
Vitamin C	50 mg	50%	75 mg	Purified L-ascorbic acid	30 mg	ND	30 mg	400 mg	30 mg	650 mg
Vitamin D	5 µg	35%	6.75 µg§	Vitamin D_3_ (cholecalciferol)	5 µg	25 µg	5 µg	50 µg	5 µg	50 µg
Vitamin E	6 mg TE	10%	6.6 mg	RRR-*α*-tocopherol acetate	0.6 mg/kg bw||	ND	6 mg||	200 mg	7 mg||	300 mg
Vitamin K	30 µg	50%	45 µg	Phylloquinone (vitamin K_1_) 5%	10 µg	ND	15 µg	ND	20 µg	ND
Zinc	10 mg	0%	10 mg	Zinc as gluconate	8.4 mg	5 mg	8.3 mg	7 mg	9.6 mg	12 mg
Iron	18 mg	0%	18 mg	Ferrous fumarate	18.6 mg	40 mg	11.6 mg	40 mg	12.6 mg	40 mg
Iodine	90 µg	0%	90 µg	Potassium iodate	90 µg	ND	90 µg	200 µg	90 µg	300 µg
Copper	340 µg	0%	340 µg	Cupric gluconate	220 µg^ 5^	ND	340 µg^ 5^	1 mg	440 µg^ 5^	3 mg
Selenium	20 µg	0%	20 µg	Sodium selenate	10 µg	60 µg	17 µg	90 µg	22 µg	150 µg
Magnesium	65 mg	0%	65 mg	Trimagnesium dicitrate anhydrous	54 mg	ND	60 mg	65 mg ¶	76 mg	110 mg ¶

RNI: Recommended Nutrient Intake as established by WHO/FAO [8]; UL: Tolerable Upper Intake Level as established by FNB/IOM [23]; RAE: retinol activity equivalents; NE: niacin equivalents; DFE: dietary folate equivalents; TE: *α*-tocopherol equivalents; ND: Not derived.

* Overage is calculated from declared amount (D) and formulated amount (F) as O = (F-D)*100/D.

† Equivalent to 1,500 IU.

‡ Based on IOM estimates that 0.5 µg folic acid taken on an empty stomach corresponds to 1 µg DFE.

§ Equivalent to 270 IU.

|| Values obtained from [18).

¶ UL applies to supplementary Mg.

### Follow-up and Case Detection

Supplementation was performed by community volunteers, each supervising approximately 20 children. Supplements were taken out of capsules, mixed with water and administered early in the morning. All volunteers reported daily to the research dispensary, and project staff followed up immediately in case a child had missed a daily supplementation dose. Parents or guardians were requested to bring study children to the dispensary when they noticed fever or when their child was otherwise unwell. A clinical officer was on permanent duty, and assessed sick children according to a standardized form. In case of reported cough or difficult breathing, the clinical officer looked for signs of respiratory distress and counted breathing frequency twice, each time for one full minute. All children received first-line treatment free of charge. When indicated, they were referred and provided with transport to the nearest district hospital. Parents could withdraw consent at any time.

Supplementation and follow-up continued until 12 March 2009, when the trial was stopped for all children simultaneously. We started the study at a later date than originally foreseen, and had to stop the trial when running out of resources, before the planned number of person-years were accrued, but after the desired number of events had been accrued.

### Outcome Definitions

We used the following pre-defined case definitions: 1) *Diarrhea*: guardian-reported loose or watery stools, with episodes separated by at least 48 h. To increase the specificity of the case definition we also defined cases as episodes as defined above with >3 loose or watery stools during a 24-h period; 2) *Acute lower respiratory infections (ALRI)*: reported cough with respiratory rate exceeding age-specific cut-off values (>50/min or >40/min for children aged 6–12 months and 12–60 months, respectively [9]); 3) *Severe pneumonia*: ALRI as defined above with one or more danger signs (lower chest in drawing, nasal flaring, grunting or head nodding) [9]; 4) *Any respiratory illness*: guardian-reported cough or difficult breathing; 5) *Fever without localizing signs*: guardian reported fever that was not accompanied by cough, diarrhea or other localizing signs and with a negative result for a malaria dipstick test; 6) *Any other illness*: guardian-reported illness with symptoms involving skin, ears, eyes and abscesses, but excluding trauma or burns. Malaria was defined by a positive result for the malaria dipstick test in children with guardian-reported fever with either a) confirmed fever (axillary temperature ≥37.5°C), or b) unconfirmed fever with inflammation (whole blood C-reactive protein concentrations ≥8 mg/L), separated by at least 14 days from a previous malaria episode. The definitions provided above are not always mutually exclusive. In those cases when illness episodes fulfilled the criteria for more than one definition, they contributed to the analyses of all of these case definitions. Children who attended the dispensary for invited follow-up visits were only counted as new episode if presenting with new symptoms.

### Laboratory Procedures

Venous blood samples were collected in tubes with EDTA and centrifuged within 2 h after collection. An aliquot was analyzed the same day by hematology analyzer (Sysmex KX21, Kobe, Japan). As a measure of inflammation, we measured whole blood C-reactive protein concentration in the field by immunoturbidimetric assay (QuikRead, Orion Diagnostica, Espoo, Finland). Plasma concentrations of zinc were determined by inductively-coupled plasma-mass spectrometry. Plasma concentrations of C-reactive protein and ferritin were measured in The Netherlands on a Beckman Coulter Unicel DxC880i system. Stool samples collected during the surveys were preserved in sodium acetate acetic acid formalin, stored at 4°C and tested for Giardia intestinalis parasites by enzyme-linked immunosorbent assay (ProSpecT Giardia Microplate Assay, ProSpecT Giardia Microplate Assay, Oxoid, Basingstoke, UK). This test has a sensitivity and specificity of 93% and 100%, respectively, as compared to detection by either microscopy or the EIA in at least one of two sequential stool samples from individual subjects [10].

#### Statistical analysis

All data were double-entered, cleaned and analyzed in SPSS (v15·0 for Windows, SPSS, Chicago, IL) and STATA (v11; College Station, Tx). Compliance was measured as the proportion of children who consumed 95% of scheduled supplements. Height-for-age z-scores were calculated based on the NCHS/WHO growth reference, using Epi Info (version 3.3.2; http://www.cdc.gov/epiinfo). Being stunted was defined as height-for-age z scores < –2 SD. New growth charts have become available for children (WHO Anthro Software, 2007; http://www.who.int/childgrowth/software/en/, [11]) since we planned our study; use of these new charts would likely have led to more children being classified as being stunted. Differences in proportions were calculated using CIA [12].

We report incidence rates, and used Cox proportional hazard models to assess intervention effects as crude and adjusted hazard ratios, as assessed in an intention-to-treat analysis. We explored effects on diarrhea and respiratory illness by analyzing cases with and without fever separately. We conducted subgroup analyses to explore potential effect-modification by age class (as defined by the strata used for randomization), being stunted, and the presence of *G. intestinalis* infection at baseline. In this analysis, we pooled children aged 18–35 months and 16–60 months because there were only 44 cases in the oldest age class. We explored to what extent adjustment for factors that were predictive for morbidity (age class, stunting, distance to the dispensary [≥ or <4 km, which is close to the median], and mosquito net use) influenced estimates of the intervention effects.

## Results

**Figure 1:**
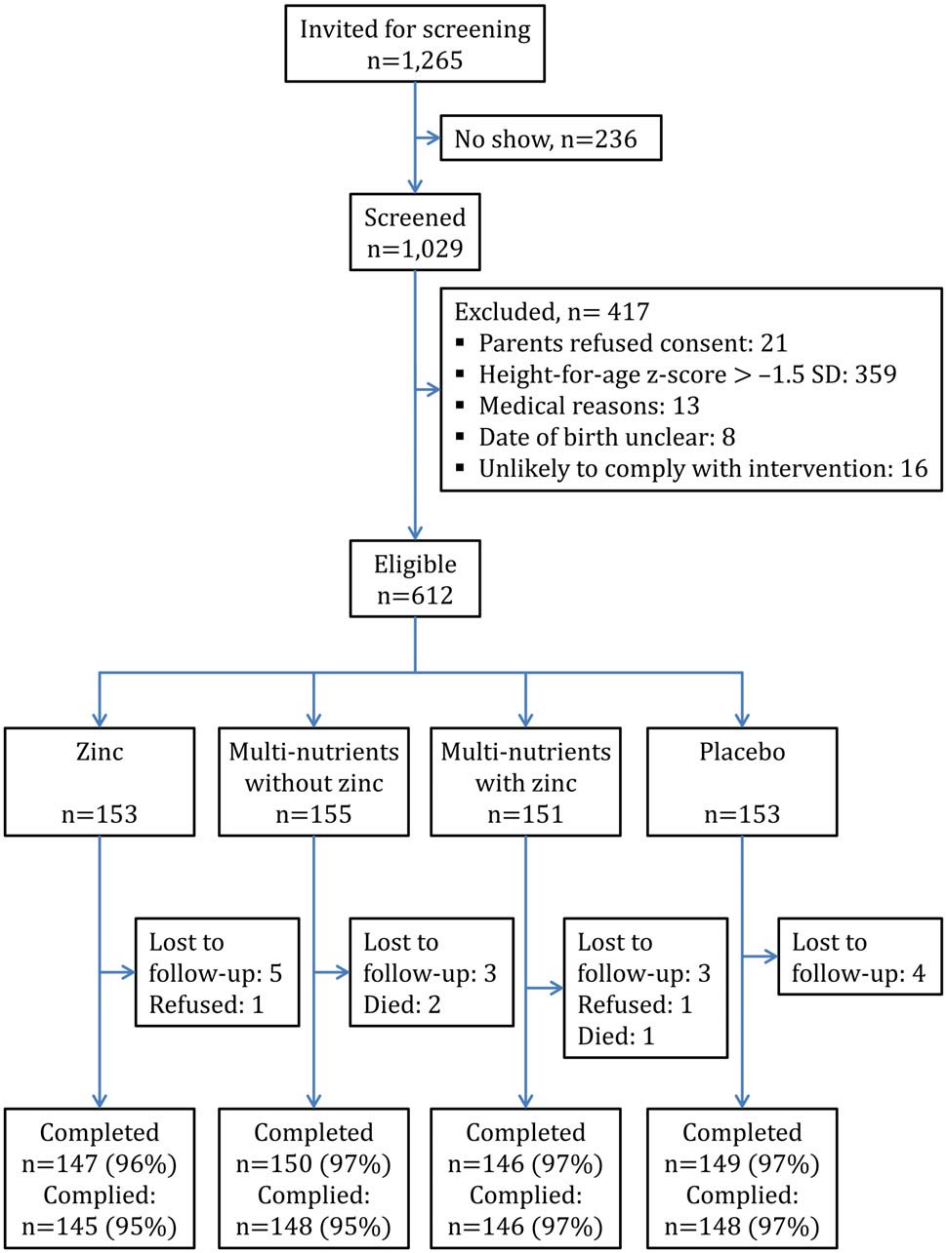
Trial profile. Compliance was measured as the proportion of children who consumed.95% of scheduled supplements.

The trial profile is shown in **Figure 1**. Baseline characteristics were similar between intervention groups (**Table 2**). The prevalence of zinc deficiency was high (67%). *Giardia* infection was detected in 192 children (31%), and 426 children (70%) were stunted.

**Table 2:** Baseline characteristics of study participants, by intervention group.

	Zinc		Multi-nutrients without zinc		Multi-nutrients with zinc		Placebo	
N	153		155		151		153	
Sex M/F [n/n]	70/83	[46%/54%]	87/68	[56%/44%]	66/85	[44%/56%]	76/77	[50%/50%]
Age	32.5	(15.4)	32.2	(15.7)	32.5	(15.5)	32.7	(16.1)
*Plasmodium* infection*	66	[43%]	64	[41%]	67	[44%]	68	[44%]
Height-for-age, z-score	–2·36	(0·69)	–2·50	(0·69)	–2·39	(0·71)	–2·45	(0·69)
Inflammation†	52	[34%]	51	[33%]	51	[34%]	47	[31%]
*Giardia intestinalis* infection‡	50	[33%]	54	[35%]	42	[28%]	46	[30%]
Zinc deficiency§	97	[63%]	110	[71%]	105	[70%]	100	[65%]
Iron deficiency||	23	[23%]	25	[24%]	24	[24%]	25	[24%]
Hemoglobin concentration, g/L	101·8	(12·6)	102·7	(12·8)	103·8	(12·7)	102.8	(12·7)
Distance from homestead to dispensary, km¶	3·66	(2·31)	3·52	(2·06)	3·54	(2·07)	3·60	(3·38)
Mosquito net use **	48	[32%]	55	[36%]	45	[30%]	46	[31%]

Mean (SD), % [n] unless indicated otherwise.

* As indicated by a positive result for pLDH-based dipstick test (see text).

† Plasma C-reactive protein concentration ≥8 mg/L.

‡ Stool samples could not be analyzed for 54 children (with 13, 14, 15 and 12 missing values in the 4 intervention groups). Prevalence calculated with total group numbers in denominator.

§ Plasma zinc concentration <9.9 µmol/L.

|| Plasma ferritin concentration <12 µg/L (6 missing values); restricted to children without inflammation at baseline (n = 101, 104, 100 and 106, respectively).

¶ Measured as the crow flies, based on global positioning data.

** Data missing for 11 children.

**Figure 2:**
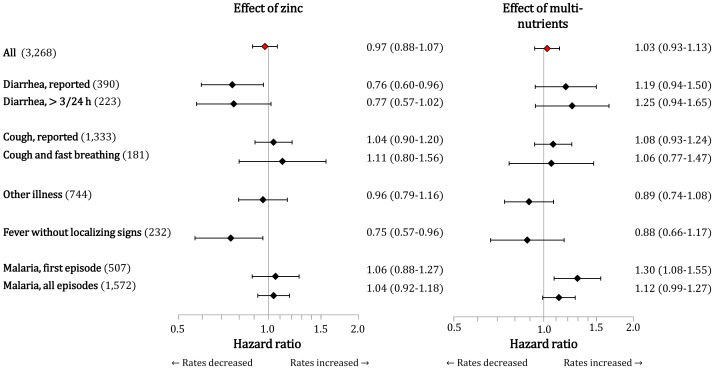
Effect of supplementation with zinc (left panel) or multi-nutrients (right panel) on selected morbidity outcomes. The effect of zinc was assessed by hazard ratios in the pooled groups receiving zinc (with or without multi-nutrients) versus the pooled groups receiving no zinc (with or without multi-nutrients); the effect of multi-nutrients was assessed by hazard ratios in the pooled groups receiving multi-nutrients versus the pooled groups receiving placebo or zinc. Values between brackets (left) indicate the number of episodes for each illness definition. Horizontal line bars indicate 95% CIs. Estimates are adjusted for age class (indicated by dummies) and distance between homestead and research dispensary (< or ≥4 km); further adjustment for mosquito net use, sex, plasma zinc concentration, iron status or inflammation at baseline did not markedly change the effect estimates.

Twenty children (3%) did not complete the trial: three died, two were withdrawn by parents, and 15 emigrated from the area (**Figure 2**). Another two children discontinued the intervention but were available for follow-up. Compliance was high (96%) and similar in all four groups.

There were 3,268 clinic visits during the study period, of which 2,462 (75%) were accompanied by guardian-reported fever and 1,572 (48%) classified as malaria. Among the non-malarial fever cases, 658 were accompanied by diarrhea, cough, or other localizing signs, either alone or in combination, while 232 fever cases were without localizing signs (**Table 3**). For 223 children (36%), parents or caretakers reported at least one episode of diarrhea. A total of 390 diarrhea episodes were recorded, with an incidence rate of 0.74/child-year. There were 1,333 episodes of reported cough, but only few (181) fulfilled criteria of ALRI as established by the World Health Organization [6]. The number of severe pneumonia cases (24) was insufficient for meaningful analysis. There were 744 visits for other reasons, mainly abscesses and symptoms involving skin, ears, and eyes.

**Table 3:** Incidence rates for various illnesses, stratified by the presence of fever.

	All		Fever reported		No fever reported	
Total observation time, child-years	526		526		526	
All clinic visits	6.2	(3,268)	4.7	(2,462)	1.5	(806)
Diarrhea						
Guardian-reported episodes	0.74	(390)	0.47	(248)*	0.27	(142)
>3 loose stools/24 h	0.44	(233)	0.41	(216)	0.03	(17)
Respiratory illness						
Cough	2.5	(1,333)	1.9	(998)*	0.64	(334)
Cough with fast breathing	0.34	(181)	0.28	(147)*	0.06	(34)
Severe pneumonia	0.05	(24)	0.04	(20)	0.01	(4)
Other illnesses †	1.4	(744)	0.68	(3,566)*	0.74	(388)
Fever without localizing signs ‡	0.44	(232)	NA		NA	
Malaria	3.0	(1,572)	NA		NA	
Hospital referrals	0.13	(68)	0.13	(68)	0.00	(0)

Values indicate the total number of episodes per child-year observed. Numbers between brackets indicate the number of episodes.

NA: Not applicable (fever is part of the case definition).

* Among these, the following number of cases was also classified as malaria: reported diarrhea: 137 (35%); reported diarrhea with ≥3 loose stools/24 h: 69 (30%); reported cough: 491 (36%); other: 166 (22%).

† Includes symptoms of skin, ears, and eyes and abscesses; excluding trauma or burns.

‡ Cases classified as fever without localizing signs are not included in any of the other categories.

Upon examination of interaction effects (**Table 4**), we found no evidence that concurrent supplementation with multi-nutrients influenced the magnitude of the effect of zinc on rates of diarrhea, respiratory illness, fever without localizing signs, or other illness. Thus in the remainder of this report, we will present marginal effects whereby the effect of multi-nutrients is assessed by comparing the pooled groups receiving placebo or zinc with the pooled groups receiving multi-nutrients, and the effect of zinc is assessed by comparing the pooled groups receiving no zinc (with or without multi-nutrients) and the pooled groups receiving zinc (with or without multi-nutrients).

**Table 4:** Incidence of various illnesses, by treatment group.

	Zinc		Multi-nutrients without zinc		Multi-nutrients with zinc		Placebo		Interaction effect*		p
Child-years at risk	130.8		133.2		129.7		131.9				
Diarrhea											
All reported episodes	0.62	(81)	0.92	(123)	0.67	(87)	0.75	(99)	0.88	(0.52–1.51)	0.65
>3 loose stools/24 h	0.35	(46)	0.56	(74)	0.42	(55)	0.44	(58)	0.96	(0.51–1.80)	0.89
Respiratory illness											
Cough	2.5	(322)	2.5	(333)	2.7	(354)	2.5	(324)	1.09	(0.80–1.48)	0.57
Cough with fast breathing	0.34	(45)	0.32	(43)	0.39	(50)	0.33	(43)	1.13	(0.57–2.24)	0.72
Other	1.5	(195)	1.4	(186)	1.3	(169)	1.5	(194)	0.92	(0.62–1.37)	0.68
Fever without localizing signs	0.39	(51)	0.45	(60)	0.37	(48)	0.55	(73)	1.17	(0.66–2.06)	0.60

Values indicate the total number of episodes per child-year observed. Numbers between brackets indicate the number of cases.

* Measured in a Cox proportional hazard model as the hazard ratio associated with the interaction term between zinc and multi-nutrients.

Zinc supplementation reduced the rate of diarrhea by 24% (95% CI: 4% to 40%) (**Figure 2**), while multi-nutrients seemed to increase rates by 19% (–6% to 50%). We found similar effects when restricting the analysis to cases of diarrhea with ≥3 loose stools/24 h (Figure 2). The protective effect appeared more evident for cases of diarrhea that were accompanied by fever (HR 0.73; 0.55–0.98) than for those not accompanied by fever (HR 0.80; 0.55–1.16), but the difference in effect was small.

There was no evident effect of either intervention on episodes of respiratory illness, whether defined as reported cough or as cough with fast breathing, or on episodes of other illnesses (Figure 2). By contrast, zinc reduced the rates of fever without localizing signs by 25% (4%–43%; adjusted for age class and distance between homestead and research clinic).

**Figure 3:**
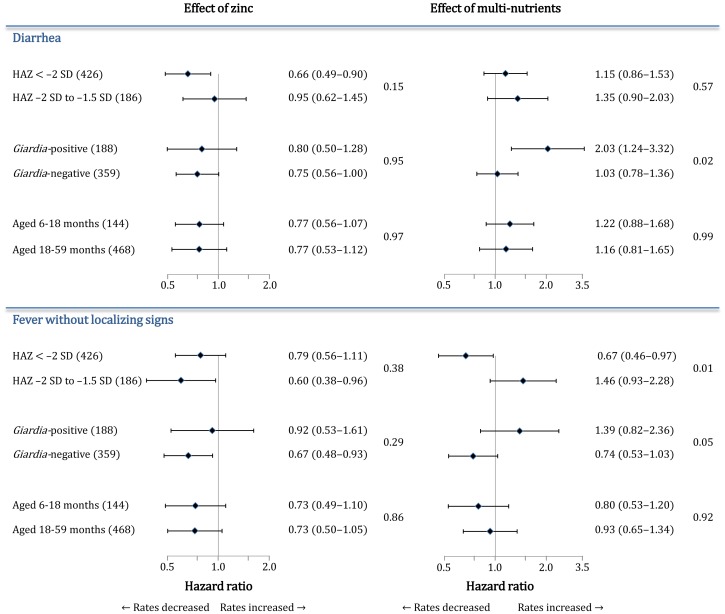
Subgroup analysis of effects of supplementation with zinc or multi-nutrients on diarrhea (top panels) and fever without localizing signs (bottom panels). Effects are indicated by hazard ratios (see Figure 2), with p-values for interaction tests. Values between brackets (left) indicate the number of children in each subgroup. Horizontal line bars indicate 95% CIs. Effects are adjusted for age class, being stunted and distance (< or ≥4 km) unless these factors were used to define subgroups. Because the presence of *Giardia* infection could not be assessed in 54 children due to missing stool samples, the analysis is based on fewer cases of diarrhea (361) and fever without localizing signs (216) than reported in the text.

Age was strongly predictive for all morbidity outcomes, but there was no evidence that age class influenced the magnitude of the effect of the interventions for any of the outcomes considered (not shown). We found weak evidence that the zinc-induced reduction in diarrhea rates was more pronounced in stunted children (0.66; 0.49–0.90) than in those with a lesser degree of stunting (0.95; 0.62–1.45; interaction effect: 0.68 (0.41–1.15); **Figure 3** **;** upper panel, left).

The effect of multi-nutrient supplementation on diarrhea depended on the presence of *Giardia* infection at baseline (Figure 3; upper panel): multi-nutrient supplementation doubled the rate of diarrhea among those with *Giardia* at baseline (2.03; 1.24–3.32), whereas it had no obvious effect among those without *Giardia* infection (1.03; 0.78–1.36). This difference in intervention effect between *Giardia*-positive and *Giardia*-negative children was independent of age and stunting, and unlikely to have occurred by chance (interaction effect: 1.98; 1.13–3.47; p = 0.02).

With regards to the effects of multi-nutrients on fever without localizing signs, patterns observed in the subgroup analysis largely resembled those observed for diarrhea (Figure 3; lower panel). Both *Giardia* infection and being stunted at baseline determined the magnitude of the effect of multi-nutrients on disease rates. Multi-nutrients resulted in increased rates (1.39; 0.82–2.36) in children with *Giardia* infection and decreased rates in those without (0.74; 0.53–1.03) (interaction effect: 1.86; 1.00–3.47). In stunted children, however, multi-nutrients decreased rates (0.67; 0.46–0.97), whereas in children with a lesser degree of stunting, multi-nutrients seemed to increase rates (1.46; 0.93–2.28; interaction effect: 0.47; 0.26–0.83).

Lastly, multi-nutrients also increased the rate of reported cough among stunted children, but not so in their less stunted counterparts (HR: 1.34; 1.06–1.69 versus 0.98; 0.82–1.17; p-value for interaction: 0.05). When analyzing effects on ALRI, a similar pattern was seen (1.41; 0.79–2.52 versus 0.99; 0.65–1.50 among stunted and less stunted children respectively), but the number of cases was lower and thus the statistical evidence for this interaction weaker (p = 0.33).

## Discussion

We found no evidence that the effect of zinc supplementation on any of the morbidity outcomes assessed was influenced by concurrent supplementation with other micronutrients. Daily supplementation with zinc reduced the rate of fever without localizing signs by approximately one-quarter. By contrast, supplementation with multi-nutrients seemed to increase the rate of diarrhea, mostly so in children with *Giardia* infection at baseline.

Many micronutrients, including zinc, are believed to play a role in innate and adaptive immune responses, notably in the proliferation and differentiation of Th1 and Th2 lymphocyte subsets in response to infection [13]. Because the effector mechanisms of these micronutrients may involve the same causal pathway, we hypothesized that an inadequate intake and deficiency would act synergistically in causing morbidity. Our findings may indicate that this hypothesis is incorrect and that such interaction truly does not exist, or the interaction effect may have been too small to allow detection in our trial.

The effect of zinc on diarrhea found in our study is consistent with the 20% reduction reported in a recent systematic review [1]. Contrary to the findings from this meta-analysis [1], however, we found no evidence that this protection depended on age class. The meta-analysis also showed that the preventive effect of zinc supplementation against diarrhea decreased with baseline height [1]. Our results suggested a similar influence by the initial degree of stunting (Figure 3) but the statistical support for such interaction was weak (p = 0.15). The incidence of diarrhea in our study was comparatively low, however, and the number of cases may have been too low to show such effect modification. For the same reason, we may have failed to show an effect of either zinc or multi-nutrients on rates of respiratory illnesses.

Based on findings from the same trial, we reported no evident effect of zinc against malaria [3]. Because they are inoculated through mosquito bites, malaria parasites bypass epithelial barriers that form the first line of defense against pathogens that cause diarrhoea and respiratory infections. The protective effect of zinc against diarrhoea, as compared to the absence of such an effect against malaria, suggests that, in our study population, zinc primarily acted by strengthening barrier function rather than through immunity.

Multi-nutrient supplementation seemed to increase diarrhea rates by 25%. This adds to findings, based on data from the same trial, that multi-nutrients increase the rate of first malaria attacks by 30% (8%–55%; figure 2), and the rate of all malaria episodes among the children aged 6–17 months and those with iron deficiency by 26% (−5% to 53%) and 41% (9%–82%), respectively [3]. Subgroup analysis suggested that multi-nutrients more than doubled diarrhea rates among children with *Giardia* infection at baseline. This finding is discussed in detail elsewhere [10].

Two other trials that investigated the added benefit of multi-nutrients in addition to zinc in preventing episodes of diarrhea also failed to show an advantage of combined supplementation above supplementation with zinc alone. In Peruvian preschool children, zinc alone tended to reduce the risk of diarrhea and respiratory illness, whereas supplementation with multi-nutrients including zinc tended to increase this risk [14]. In South African children, there was no evidence that supplementation with multi-nutrients including zinc was more efficacious than zinc alone in reducing the burden of diarrhea or respiratory illnesses [15], [16]. Taken together, these findings do not support and even caution against multi-nutrient supplementation in areas that are endemic for malaria and other infectious diseases.

*Giardia* infection at baseline influenced the effect of multi-nutrients on rates of fever without localizing signs in a similar pattern as the effect on diarrhea, even when adjusting for age and distance between homestead and research clinic. Although our definition of fever cases without localizing signs excluded cases of diarrhea, this similarity in effect patterns (including the fact that zinc protected against both outcomes) suggests that many of these fever episodes may have been due to enteric infections. Further studies are needed to establish whether *Giardia* infection caused this effect modification or whether it was a marker of an unknown factor that may have played such a role, and to identify the mechanisms involved.

Our study design did not allow us to identify single nutrients that may have caused the increase in diarrhea associated with the multi-nutrient supplement among children with *Giardia*. At the doses provided, it is unlikely that magnesium or vitamin C caused osmotic diarrhea; we also found no evidence, which otherwise might have been expected, that the increased diarrhea rates were larger in the youngest children who received a larger dose per body weight that older children (Figure 3). Whilst most micronutrients seem to protect against diarrhea [17], supplementation with iron may increase the diarrhea incidence [18], possibly by enhancing proliferation and virulence of enteric pathogens, or facilitating pathogen invasion by increasing permeability of the small intestine [19], [20]. Iron may also influence or impair the immune responses to pathogens [17]. *In vitro* studies using Caco-2 cells suggest that copper may also impair intestinal barrier function by enhancing paracellular permeability [21], [22].

We found no evidence that the addition of vitamins and other minerals to zinc supplements is helpful in preventing morbidity; in fact, it may increase the diarrhea rates in specific subgroups. This study adds support that zinc supplementation reduces the risk of diarrhea in African children and, although it did not reduce the overall number of clinic visits, it may reduce inappropriate use of anti-malarial drugs and antibiotics by reducing rates of fever without localizing signs. Our results should therefore encourage efforts to increase the intake of zinc in vulnerable populations.

## Supporting Information

Checklist S1 **CONSORT checklist.**
(DOC)

Protocol S1 **Trial protocol.**
(DOC)
